# A comparison of diabetic smokers and non-smokers who undergo lower extremity amputation: a retrospective review of 112 patients

**DOI:** 10.3402/dfa.v3i0.19178

**Published:** 2012-10-16

**Authors:** J. Joseph Anderson, Joshua Boone, Myron Hansen, Loren Spencer, Zflan Fowler

**Affiliations:** 1 Alamogordo Orthopaedics & Sports Medicine, Alamogordo, NM; 2 Scripps Mercy Hospital, San Diego, CA; 3 Cactus Foot & Ankle, LLC, Chandler, AZ

**Keywords:** diabetes, smoking, amputation, foot, lower extremity

## Abstract

**Background:**

A diabetic foot or lower extremity amputation may be exacerbated by or related to the smoking habits and history of the patient.

**Patients and methods:**

Of the 112 diabetic patients in this retrospective study, 46 were non-smokers and 66 were smokers. The smokers were further categorized into patients who: 1) did not cease smoking; 2) ceased in the immediate post-operative period but resumed within 3 months; and 3) ceased up to and at the 3-month post-operative period. The patients were also divided by their amputation level of forefoot, midfoot/rearfoot, and proximal leg.

**Results:**

Smoking diabetic patients underwent more amputations, as well as more proximal amputations than those who did not smoke. The higher amount of smoking in pack years followed an increasing trend of more proximal amputations as well.

**Conclusion:**

Neither the amputation level nor the amputation itself was enough motivation for the patients to participate in smoking cessation.

It is well-established that cigarette smoking has numerous harmful effects on the human body. These negative effects also extend into the realm of foot and ankle surgery. Cigarette smoking is known to increase the risk of diabetes and peripheral arterial disease (PAD), and it also delays surgical healing in both elective and emergent diabetic foot surgery. The complications in bone healing are well documented in the smoking populations (1). Lower extremity amputations are often an unfortunate outcome for someone who develops a foot ulcer (2). Despite public health efforts, the incidence of amputations in the diabetic population continues to increase. The 5-year mortality rate in diabetics who underwent lower extremity amputation, particularly below the knee amputations (BKA), has been reported to be as high as 50% (3). There is also a significantly increased functional disability in diabetics who underwent lower extremity amputation when compared to those who did not (4).

It is well known that tobacco use increases the risk of amputation. Liedberg and Persson demonstrated that out of 188 lower limb amputees in Sweden only 23 were not diabetics, smokers, or 80 years of age or more (5). Smoking has been shown to delay healing in patients undergoing transmetatarsal amputations (TMA). A smoking history of >20 years was one key factor that was shown to lead to a more proximal amputation in 40% of those who underwent a TMA (6). There are several ways in which cigarette smoking inhibits tissue healing. Tissue perfusion and oxygenation are decreased by harmful by-products of cigarette smoke such as carbon monoxide and hydrogen cyanide that inhibit the normal metabolism of healing (7). Further, tissue perfusion may be decreased as nicotine stimulates epinephrine and norepinephrine release. Nicotine may also affect osteoblast directly and this prevents bone healing (8).

In our retrospective study, we explored the relationship between smoking and amputations in a diabetic cohort. First, we were interested to find whether diabetics who smoked had more amputations than non-smokers and also whether diabetics who smoked underwent more proximal amputation than those who did not. Second, this study will comment on the post-operative smoking habits that diabetics demonstrated after a lower extremity amputation. Finally, the post-operative effect of lower extremity amputation on the diabetic patients’ smoking habits was examined.

## Methods

One hundred and twelve diabetic patients underwent a lower extremity amputation from July 2002 to July 2010 in the practice of the primary author. Inclusion criteria included patients with a follow up of greater than 3 months after the amputation and history of diabetes. Exclusion criteria consisted of mortality occurrence within the perioperative course and less than 3 months of patient follow up. Information on current smoking history, including number of pack years, was collected. Patients were followed during the post-operative course for at least 3 months and at each subsequent office visit their current smoking status was enquired. The level of the amputation was also noted. The study was reviewed and approved by institutional review board.

Patients were divided into two groups in regards to their smoking history. Patients were defined as smokers if they had ≥5 pack years of smoking history and/or were smoking at the time of amputation. Non-smokers were defined as patients who had never smoked or had a smoking history of ≤5 years and were not currently smoking at the time of the surgery. Patients were also divided into three groups based on the level of amputation. The first group, forefoot, included all amputations distal to the midshaft of the metatarsals. Second, the midfoot/rearfoot group, included TMA and amputations through Lisfranc's, Chopart's, or ankle joint. The final group was defined as proximal leg to include all BKA and above the knee amputations (AKA). Some reasons for amputations in the diabetic patients were poor blood flow, usually in combination with neuropathy or infections like gangrene.

There were 66 smokers and 46 non-smokers in our study; of these, 68 were males and 44 were females. There were 69 distal amputations, 32 midfoot/rearfoot amputations, and 11 proximal leg amputations for a total of 112 amputations.

The smoking habits of the diabetic smokers (defined in our study as ≥5 pack years of smoking) were examined during the perioperative course. Smoking habits during the perioperative course were divided into three categories. First, those who ceased smoking for at least 3 months, second those who ceased smoking initially but resumed within 3 months of their amputation, and finally, those who never ceased smoking. The post-operative effects of lower extremity amputation on the diabetic patients’ smoking habits were also examined.

## Results

A total of 112 patients fit our inclusion criteria. The average age of the patients at the time of the amputation was 61.5 years. There were 66 smokers and 46 non-smokers. The average number of pack years in the smoking group was 33.8. The demographic data are represented in Table 1. The number of diabetic smokers who underwent lower extremity amputation was compared to their non-smoking counterparts in regard to incidence and age at the time of amputation and the results showed a significantly greater number of amputations in diabetic smokers than the non-smokers (*p*=0.038).

**Table 1:** Demographic description of cohort (N=112)

Age at operation (years)	61.5 (23 to 92)
Male/female	61%/39% (68/44)
Smokers/non-smokers	59%/41% (66/46)*
Average pack years of smoking	33.8 (5–100)

* *p*=0.038

The levels of amputation in 66 smokers who underwent lower extremity amputation, further examined and subdivided into 37 forefoot, 19 midfoot/rearfoot, and 10 proximal leg amputations, were compared to 46 non-smokers, subdivided into 32 forefoot, 13 midfoot/rearfoot, and 1 proximal leg amputation (Table 2). There was a statistically significant difference in the levels of amputation between smokers and non-smokers (*p*=0.031). Of the 11 patients who underwent proximal leg amputations, 10 were smokers while only 1 was a non-smoker. Table 3 demonstrates the number of pack years for smokers who underwent one of the three levels of amputations and though not statistically significant (*p*=0.315), there was a trend toward an increase in the number of average pack years as the amputation level became more proximal.

**Table 2:** Comparison of the level of amputation in smokers and non-smokers

	Smokers	Non-smokers
Amputation level		
Distal	37	32
Midfoot/rearfoot	19	13
Proximal leg	10	1

*p*=0.031.

**Table 3:** Number of pack years in diabetic smokers in relationship to level of amputation

Amputation level	Amputation numbers	Number of pack years
Distal	37	32.5
Midfoot/rearfoot	19	33.3
Proximal leg	10	40.3

*p*=0.492.

The authors also evaluated whether the smoking group was likely to change their smoking habits after a lower extremity amputation. Furthermore, we examined if there was any correlation between level of amputation and change in smoking habits. This data is represented in Table 4. Of the smokers who had a distal amputation, 16 never ceased smoking, 13 ceased but restarted within 3 months, and 8 ceased for >3 months. In those with midfoot/rearfoot amputations, nine, eight, and two were the number of patients for the smoking habits of never ceased, ceased, but restarted and had ceased at 3 months, respectively. These data were not statistically significant (*p*=0.069) and suggest that there was minimal change in smoking habits despite undergoing a lower extremity amputation.

**Table 4:** The effects of lower extremity amputations on diabetic smoking habits status after amputation

Amputation level	Never ceased	Ceased and started	Ceased > 3 months
Distal	16	13	8
Midfoot/rearfoot	9	8	2
Proximal leg	5	2	3

*p*=0.455.

## Discussion

Lower extremity amputation continues to be an unfortunate complication in patients with diabetes. In addition to functional limitations that amputees experience, there is also a significant psychological impact (9). Depression has been shown to increase the number of macro- and microvascular complications in diabetics (10). Recently, comorbid depression in diabetics has been shown to increase mortality, excluding cardiovascular complications caused by depression (11).

One finding from our data shows that diabetic patients who smoke are more likely to undergo amputation than the non-smokers (*p*=0.038). This information is important to note and to relay to patients who continue to smoke while having diabetes. Another of several reasons to relay the importance of cessation is the increased risk of PAD. Also of note were patients who underwent a BKA. Of the 11 patients subjected to BKA, 10 were smokers. There was a significant trend toward diabetic smokers requiring a more proximal amputation than non-smoking diabetics.

Our retrospective review demonstrated that amputation did not appear to affect the smoking habits of diabetics. As seen in Table 1, the average number of pack years for the subject population was 33.8 years. This is surprisingly high and our sample size may have been biased to those with a high number of pack year histories. However, undergoing an amputation did not encourage cessation of smoking in an individual.

There are several limitations in this study. Limitations include variables such as PAD, neuropathy, hemoglobin A1C, and body mass index. However, this study was performed to examine one particular correlation between smoking and amputation, and it is unknown how these other variables would have affected our results. Study weaknesses include its retrospective design, the lack of blood work on patients to confirm nicotine levels and short follow up of 3 months, making it unclear if those who were able to cease smoking were able to continue this long term. Also, our information on patients’ smoking habits was based on their responses. The information we received should be fairly accurate, but there is a possibility of it being false or misleading data based on the patient's statements, or there was reason for doubt, for example, the patient smelled strongly of tobacco smoke, family members were also questioned and patient's answer changed when asked again.

Aiding patients in smoking cessation is a challenge faced by all medical practitioners. While the primary medical provider typically coordinates smoking cessation efforts, foot and ankle surgeons can play an important role in the process. Reviewing and explaining individualized patient- and diagnosis-specific information is one way that has shown to be an effective aid in smoking cessation (12). It is difficult to predict what adverse outcomes smokers will have if they smoke before and after the procedure. However, specific examples of the negative impact on bone, wound healing, and more proximal amputation if smoking continues may be helpful (1).

The overall conclusion is that diabetic patients who smoke are more likely to undergo a lower extremity amputation when compared to diabetic non-smokers. Also, though not statistically significant, there is a trend toward diabetic smokers receiving a more proximal amputation when compared to their non-smoking counterparts. Educating patients on the increased risk of amputations with continued smoking and diabetes may play a role in smoking cessation.
